# Down-Regulation of HtrA1 Activates the Epithelial-Mesenchymal Transition and ATM DNA Damage Response Pathways

**DOI:** 10.1371/journal.pone.0039446

**Published:** 2012-06-26

**Authors:** Ning Wang, Kristin A. Eckert, Ali R. Zomorrodi, Ping Xin, Weihua Pan, Debra A. Shearer, Judith Weisz, Costas D. Maranus, Gary A. Clawson

**Affiliations:** 1 Gittlen Cancer Research Institute & Department of Pathology, College of Medicine, Pennsylvania State University, Hershey, Pennsylvania, United States of America; 2 Department of Chemical Engineering, Pennsylvania State University, University Park, Pennsylvania, United States of America; 3 Department of Obstetrics & Gynecology, Pennsylvania State University, Hershey, Pennsylvania, United States of America; 4 Materials Research Institute, Pennsylvania State University, University Park, Pennsylvania, United States of America; University of Nebraska Medical Center, United States of America

## Abstract

Expression of the serine protease HtrA1 is decreased or abrogated in a variety of human primary cancers, and higher levels of HtrA1 expression are directly related to better response to chemotherapeutics. However, the precise mechanisms leading to HtrA1 down regulation during malignant transformation are unclear. To investigate HtrA1 gene regulation in breast cancer, we characterized expression in primary breast tissues and seven human breast epithelial cell lines, including two non-tumorigenic cell lines. In human breast tissues, HtrA1 expression was prominent in normal ductal glands. In DCIS and in invasive cancers, HtrA1 expression was greatly reduced or lost entirely. HtrA1 staining was also reduced in all of the human breast cancer cell lines, compared with the normal tissue and non-tumorigenic cell line controls. Loss of HtrA1 gene expression was attributable primarily to epigenetic silencing mechanisms, with different mechanisms operative in the various cell lines. To mechanistically examine the functional consequences of HtrA1 loss, we stably reduced and/or overexpressed HtrA1 in the non-tumorigenic MCF10A cell line. Reduction of HtrA1 levels resulted in the epithelial-to-mesenchymal transition with acquisition of mesenchymal phenotypic characteristics, including increased growth rate, migration, and invasion, as well as expression of mesenchymal biomarkers. A concomitant decrease in expression of epithelial biomarkers and all microRNA 200 family members was also observed. Moreover, reduction of HtrA1 expression resulted in activation of the ATM and DNA damage response, whereas overexpression of HtrA1 prevented this activation. Collectively, these results suggest that HtrA1 may function as a tumor suppressor by controlling the epithelial-to-mesenchymal transition, and may function in chemotherapeutic responsiveness by mediating DNA damage response pathways.

## Introduction

HtrA1 (also referred to as Prss11, or IGFBP-5) is a member of the High Temperature Requirement Factor A (HtrA) family of oxidative stress-response proteases. The human HtrA1 gene was initially identified as being expressed in normal human fibroblasts, but not after their transformation with SV40 [1]. HtrA1 is expressed as an Mr 51,000 precursor with a signal sequence, which presumably targets some HtrA1 for secretion. However, we have also observed intracellular (cytoplasmic and nuclear) HtrA1 forms in a variety of epithelial cell types, as well as an Mr 29,000 intranuclear proteolytically active form [2]. HtrA1 is ubiquitously expressed in normal human tissues; for example, De Luca et al. documented high HtrA1 expression levels in mature layers of epidermis, in secretory breast epithelium, in liver, and in tubules of the renal cortex [3], suggesting it may have many functions. In fact, HtrA1 has been implicated in diverse diseases, particularly age-related macular degeneration [4] and nervous system arteriopathies (CARASIL; see [5]).

A number of studies have suggested that HtrA1 may function as a tumor suppressor. HtrA1 has been reported to be absent or substantially down-regulated in a variety of cancers during their progression, including gastric, breast, ovarian [6], endometrial [7] and hepatocellular [8] carcinomas, as well as mesothelioma [9] and melanoma [10]. Down-regulation of HtrA1 expression in the ovarian cancer cell line SKOV3 promoted cell anchorage-independent growth, while over-expression of HtrA1 in another ovarian cancer cell line OV2O2 induced cell death [11]. A similar down-regulation of HtrA1 was observed in melanomas, and over-expression of HtrA1 inhibited cell proliferation in vivo in a mouse model [10].

Modulation of HtrA protein levels may have relevance for cancer therapy, as supported by a number of findings in animal models and human cancers [12], [13]. *First,* HtrA1 is involved in estrogen-induced nephrocarcinogenesis in Syrian hamsters [14]. Within the first 5 h of estrogen treatment, HtrA1 RNA and protein increased significantly, presumably as a result of the induced oxidative stress. However, during prolonged estrogenization and cancer development, significant reductions in HtrA1 RNA and protein were observed [15]. *Second,* HtrA1 expression in human ovarian cancers was significantly decreased compared with normal ovary or with benign ovarian neoplasms [14]. *Third,* HtrA1 RNA and protein expression was decreased in human endometrial cancers vs. normal controls [16], [17], with significant negative correlations between HtrA1 and TGFβ1 levels [16], and HtrA1 protein expression and endometrial cancer grade [17]. *Fourth,* positive, statistically significant relationships have been found between HtrA1 expression level and survival in patients with gastric cancer [18] and mesothelioma [9]. *Fifth,* overexpression of HtrA1 in a metastasis-competent melanoma cell line strongly inhibited proliferation and invasive capability, and reduced HtrA1 expression was related to progression of melanomas in patient samples [10]. Despite these correlations, the tumor suppressor function(s) of HtrA1 has not yet been definitively tested or proven in animal models.

In addition to its potential role as a tumor suppressor, HtrA1 also has been implicated in chemotherapeutic responsiveness. Folgueira et al. identified HtrA1 as one of a cohort of only 3 genes (HtrA1, MTSS1, CLPTM1) that could distinguish doxorubicin-responsive from non-responsive breast cancers in 95% of samples [19]. Chien and co-workers [11] showed that HtrA1 expression enhanced sensitivity to cisplatin and paclitaxel, whereas down-regulation attenuated cytotoxicity. Down-regulation of HtrA1 was associated with resistance to apoptosis [20]. Importantly, the induction of apoptosis by HtrA1 was dependent upon its protease activity [11]. Expression levels of HtrA1 in patients with ovarian or gastric cancers correlated with their response rate to cisplatin-based treatment regimens [6], [18]. All of these findings suggest that down-regulation of HtrA1 plays an essential role in resistance to chemotherapy.

Functionally, HtrA1 inhibits cell migration via association with microtubules [21], and tubulins are known HtrA1 substrates [22]. These data are provocative, as intermediate filaments are recognized as an important target for oxidative damage [23], [24] and certain chemotherapeutics. Recent data suggest that the epithelial-mesenchymal transition (EMT) may play a critical role in the regulation of drug resistance [25]–[29]. For example, higher E-cadherin expression in cancers cells correlates with greater sensitivity to EGFR kinase inhibitors, while mesenchymal-like cells are more drug-resistant [30]. Cancer cells can also undergo adaptive changes after therapy to develop drug resistance that may involve programs like the EMT [31]. To date, the mechanisms that can induce the EMT involve multiple extracellular triggers and intracellular signaling pathways [32], [33], [34]. Deregulation of the response to reactive oxygen species (ROS) also has been related to the EMT [25].

Here, we examined HtrA1 expression in human breast specimens, including “normal” ductal epithelium, ductal carcinoma in situ (DCIS) and invasive cancers. Normal ductal epithelium displays a spectrum of intensity of immunohistochemical (IHC) staining for 4-hydroxynonenal, a marker indicative of oxidative stress [35]. Normal ductal epithelium routinely showed strong staining for HtrA1, with characteristic patterns of staining. In marked contrast, DCIS and invasive cancers showed greatly reduced or abrogated expression of HtrA1. Using the immortalized MCF10A model, we created stable cell lines with major reductions in HtrA1 expression and a stable cell line over-expressing HtrA1. Using these cell lines, we examined the effects of altered HtrA1 expression levels using gene expression and microRNA (miR) arrays. We observed significant alterations of genes involved in modulating the EMT phenotype, and validated the EMT changes by several approaches, including EMT biomarkers and cellular phenotypic properties. We also observed effects of HtrA1 expression levels on genes involved in the DNA damage response, and assessed functional changes in ATM-regulated proteins following acute oxidative stress as a consequence of HtrA1 expression levels. These results provide clues regarding the seemingly disparate roles of HtrA1 as both a putative tumor suppressor and as a modulator of chemotherapeutic responsiveness.

## Results

### Characterization of HtrA1 in Human Breast Cancers and Breast Epithelial Cell Lines

**Figure 1:**
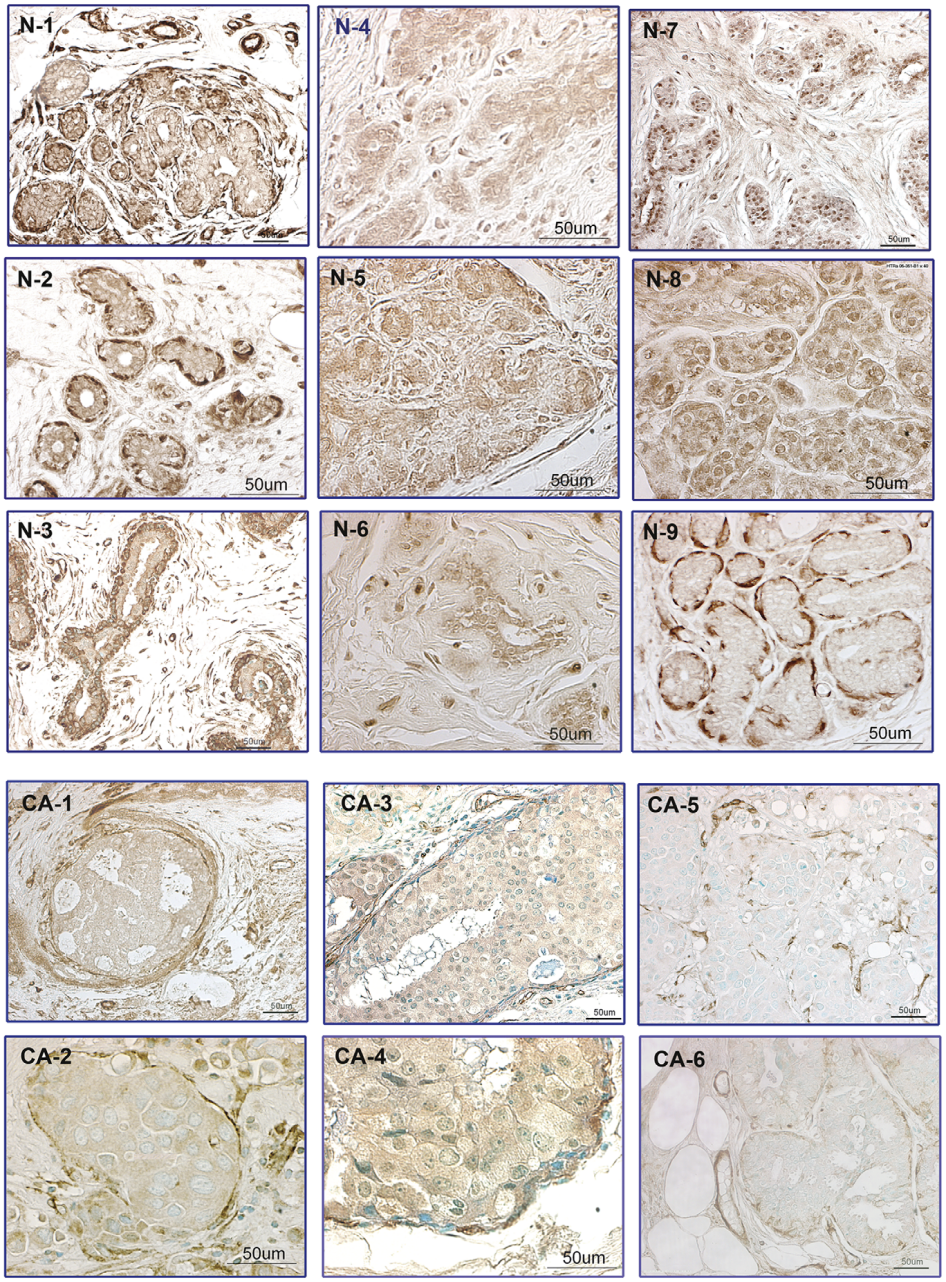
Immunohistochemical Staining of Human Breast Tissues for HtrA1. Breast tissue specimens were obtained at reduction mammoplasties (from eight subjects used as normal controls, Panels N-1 through N-9), and from mastectomies from five patients with breast cancer (Panels CA-1 through CA-6). Sections from controls were not counterstained, while sections from breast cancers were counterstained with Vector™ methylgreen, due to the greatly diminished immunostaining observed for HtrA1 in DCIS/cancers. Images were captured using a Nikon Eclipse E600 microscope with Nomarski optics and a Spot Digital Camera (Diagnostic Instruments, Inc.) with Image Pro Plus software (version 2). In normal controls there was consistent immunostaining for HtrA1, albeit with heterogeneity both in the intensity and subcellular localization of immunostaining. Panels N-1 through N-9 illustrate the three basic patterns of subcellular localization of immunostaining of epithelial cells in mammary ducts in normal controls; 1) Immunostaining of both the basal and luminal epithelial cells (N-1 through N-3); 2) Predominant immunostaining in luminal epithelial cells (N-4 through N-6), often with prominent nuclear immunostaining (N-7 & N-8); and 3) Immunostaining only of basal myoepithelial cells (N-9). These patterns may reflect oxidative stress in different microenvironments (35). Panels CA-1 through CA-6 illustrate the decreased HtrA1 immunostaining in the DCIS components of invasive breast cancers (CA-1 through CA-4), and complete loss of HtrA1 immunostaining in invasive components of breast cancers (CA-5 and CA-6) which we characteristically observed. Panels CA-2 and CA-3 show an intraductal DCIS lesion photographed at low (20X) and high (40X) power, respectively. Note that in DCIS immunostaining of the basal layer (although fragmented) is present. Control sections incubated with cocktail from which the primary HtrA1 antibody was omitted were uniformly immunonegative. Bars indicate 50 µm, as indicated.

We began by examining human breast cancer specimens using IHC. For initial studies, 3 examined 3 antibody preparations for HtrA1. We observed a relatively high background staining with the polyclonal antisera preparation which has been widely used for previous studies on HtrA1. We therefore obtained 2 affinity purified HtrA1 antibodies; both showed similar staining patterns, although staining was uniformly stronger with one of them (from Sigma), which was used for subsequent studies. Normal ductal epithelium showed strong IHC staining for HtrA1, which manifested in different staining patterns within the same tissue specimens. One characteristic staining pattern showed prominent nuclear staining in ductal epithelial cells (Figure 1, panels N7 & N8), whereas other glands showed balanced cytoplasmic + nuclear staining (Figure 1, panels N1–N3). A third less common pattern often showed prominent HtrA1 staining within the myoepithelial cells in basement membrane Figure 1, panel N9), as well as in blood vessels. In DCIS and invasive cancers, HtrA1 expression was greatly reduced or lost entirely (Figure 1, panels CA1–CA6). Vimentin (VIM) staining in DCIS and invasive cancers was quite variable, with occasional glands showing strong epithelial staining focally localized to the basolateral regions (data not shown).

**Figure 2:**
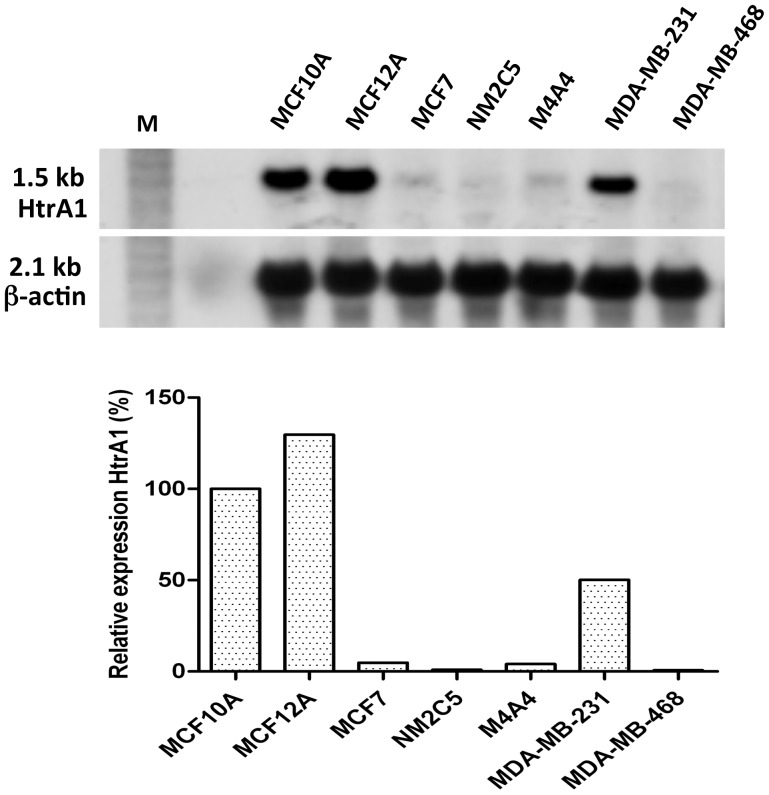
HtrA1 Gene Expression in hBC cell lines. RNA was isolated from the various human breast epithelial cell lines and expression levels of HtrA1 mRNA were determined using QPCR (Lower Panel) and Northern blot analyses (Upper Panel) as described. Results are representative of multiple independent analyses. Expression levels were 20–25X higher in the non-tumorigenic MCF10A and 12A cell lines, with very low expression levels in most of the hBC cell lines (MDA-MB-231 was the exception; see text). β-actin transcript were used as to assess loading on Northern blots.

**Figure 3:**
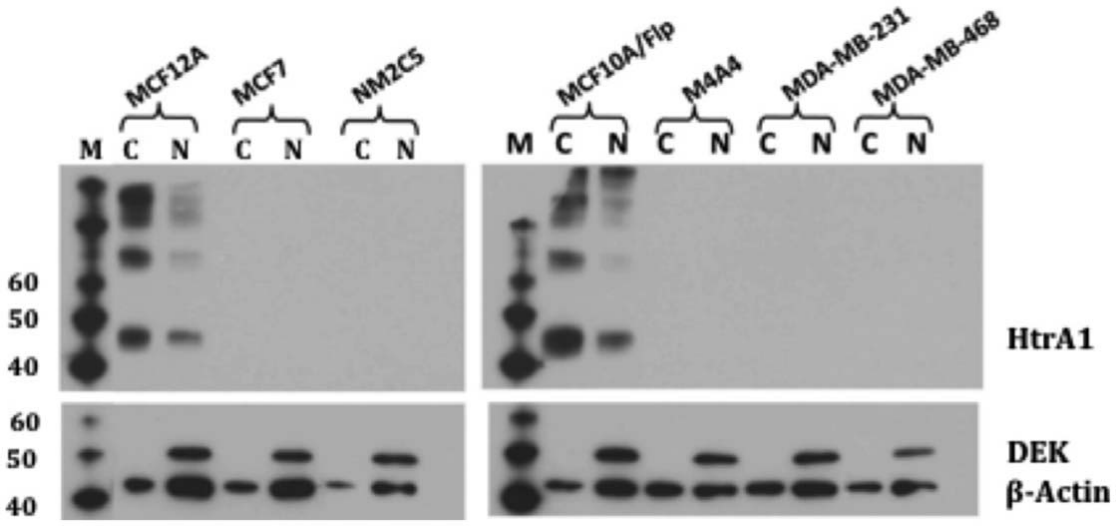
HtrA1 Protein Expression in hBC cell lines. Cells (as indicated) were extracted into nuclear (N) and cytoplasmic (C) fractions as described (Materials & Methods), and proteins were analyzed by Immunoblot analyses using the polyclonal antibody against human HtrA1. β-actin was used as a loading control, and DEK was used to assess the nuclear/cytoplasmic fractionation (DEK is exclusively nuclear). The larger Mr bands seen in the MCF12A and MCF10A/Flp cell lines are consistent with dimers, trimers, etc., although this was not confirmed. MCF10A/Flp is the parental Flp-in cell line which was used to produce the various MCF10A/siRNA or Htra1 cell lines. Results are from a representative experiment.

We next examined HtrA1 transcript levels in 7 breast epithelial cell lines, including 5 human breast cancer (hBC) cell lines (MCF7, MDA-MB-231, MDA-MB-468, NM2C5, and M4A4), and 2 immortalized non-tumorigenic cell lines (MCF10A and MCF12A), by QPCR. HtrA1 gene expression was dramatically decreased in hBC cells compared to their non-tumorigenic counterparts (Figure 2). The differences were generally greater than 20X (P<0.005), except for the MDA-MB-231 cell line, which showed HtrA1 mRNA levels which were ∼50% of those found in MCF10A cells. We further confirmed the expression differences by Northern blot analysis (Figure 2), which showed a single transcript. Immunoblot analyses demonstrated that HtrA1 protein expression was high in the non-tumorigenic MCF12A and MCF10A cell lines, but undetectable in all of the hBC cell lines tested (Figure 3). Results from the MDA-MD-231 cell line indicate a translational block, since HtrA1 mRNA is relatively high but the protein is absent.

**Figure 4:**
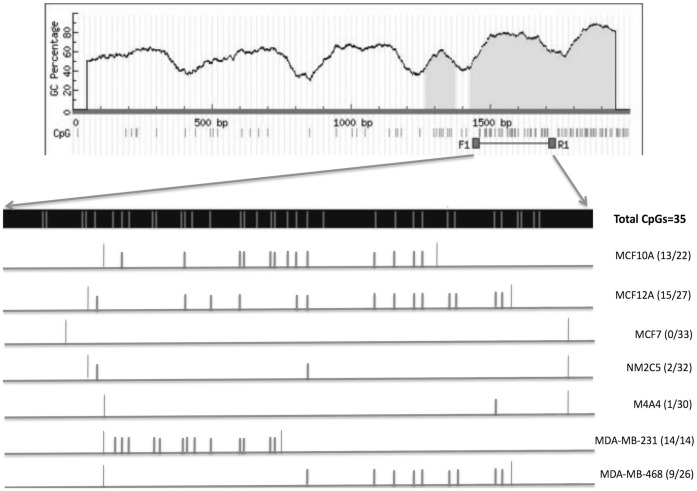
Promoter Methylation Status in the MCF10A cell lines. Candidate CpG islands were identified in the HtrA1 promoter sequence, within the upstream region from bp −561 to −266. DNA was extracted from the various cell lines and analyzed via bisulfite sequencing. The topmost Panel shows this region of interest, which is expanded in the dark strip from F1–R1, with CpG sites indicated by vertical lines (the total of 35 CpGs are marked). Unmethylated CpGs identified by bisulfite sequencing are depicted in the lower panels for the various cell lines, as indicated on the right.

Loss of gene expression could arise from several mechanisms, including genetic and epigenetic changes. We examined the cell lines for genetic defects in HtrA1. Analysis of genomic DNA did not show any loss of HtrA1, and DNA sequence analysis of the 1.5 kbp cDNAs produced from expressed transcripts revealed no point mutations in any of the cell lines (data not shown). To test potential epigenetic mechanisms, we examined HtrA1 promoter methylation status, focusing on the 800bp upstream of the HtrA1 transcription start site, which is GC-rich and includes two CpG islands within the −561 to −266 bp region. Using bisulfite gene sequencing, we observed an inverse correlation between mRNA levels and DNA methylation status from within this region, which encompasses a total of 35 CpGs (Figure 4). In MCF10A and MCF12A cells, ∼60% of the CpGs were found to be unmethylated (Figure 4). In contrast, of the CpGs examined in the tumorigenic cell lines, only 3–6% were unmethylated in the NM2C5 and M4A4 cell lines, and no unmethylated CpGs were detected in the MCF7 cell line. These results suggest that the decreased expression of HtrA1 in these cell lines may be caused by promoter hypermethylation. All CpGs in the 14 total analyzed (from the more distal CpG island, −501 to −415 bp) in the MDA-MB-231 cell line were unmethylated (Figure 4). However, we were unable to sequence the more proximal CpG island in the MDA-MB-231 cell line for unclear reasons, although the PCR product was the same size as those from the other cell lines. From the results, the more distal CpG sites appear to have greater importance in silencing of HtrA1 expression (for example, compare the MDDA-MB-231 profile to that of MDA-MB-468).

**Figure 5:**
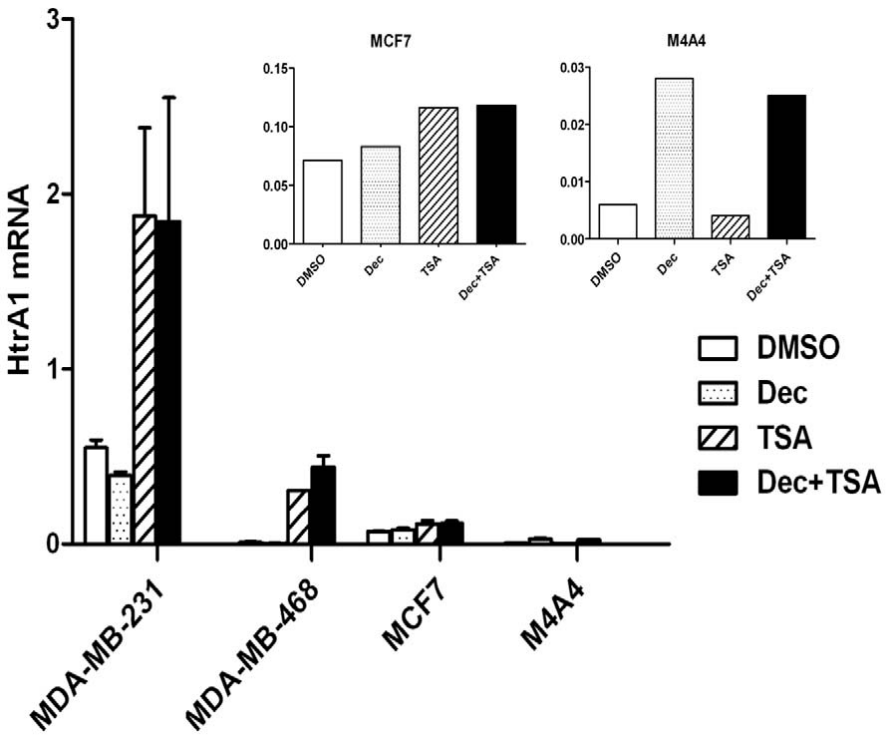
Effects of DNA methyltransferase and histone deacetylase inhibitors on HtrA1 transcript levels in human breast cancer cell lines. The indicated cell lines were treated with the DNA methyltransferase inhibitor decitabine (DEC) and/or with the histone deacetylase inhibitor TSA, as described, and resultant effects on HtrA1 transcript levels were determined using QPCR. Values ± SEs are shown from a representative experiment, which was repeated 2 times. Values for Dec and TSA were significant at p<0.01 for the MDA-MB-231 and −468 cell lines. Inset shows an expanded view of the MCF7 and M4A4 results.

Histone deacetylation is another possible epigenetic mechanism for down-regulation of HtrA1 gene expression. There is some supportive evidence for this mechanism provided by the studies of Zupkovitz et al., which reported that the mouse HtrA1 gene was one of those negatively regulated by mouse histone deacetylase 1 (HDAC1) [36]. We used decitabine (DEC), a DNA methyltransferase inhibitor, and trichostatin A (TSA), a histone deacetylase inhibitor, to characterize the role of DNA methylation and histone deacetylation in controlling HtrA1 gene expression. Four hBC cell lines (MDA-MB-231, MDA-MB-468, MCF7, and M4A4) were treated with DMSO (as a solvent control), 5 µM DEC, 300 nM TSA, or DEC + TSA in combination for 72 hr. After the treatment, RNA was harvested and subjected to QPCR analysis for HtrA1 (Figure 5). Two of the cell lines showing complete promoter methylation (MCF7 and M4A4) showed little response to either inhibitor (with M4A4 cells, the small response was attributable to DEC alone). However, the 2 cell lines which showed only partial promoter methylation (MDA-MB-231 and MDA-MB-468) showed highly significant (p<0.01) increases in HtrA1 expression levels. These results indicated that HtrA1 expression in MDA-MB-231 and MDA-MB-468 cell lines was inhibited (albeit to different extents) by histone deacetylation, whereas the gene silencing in MCF7 and M4A4 cells was due largely to DNA hypermethylation. In the MCF7 cell line, DNA methylation and histone deacetylation may be cooperating in down-regulation of HtrA1 gene expression. Overall, the results demonstrate that HtrA1 is consistently down-regulated in all hBC cell lines, and that the down-regulation is due to various epigenetic mechanisms.

#### Mechanistic studies of HtrA1 function(s) in MCF10A cells

**Figure 6:**
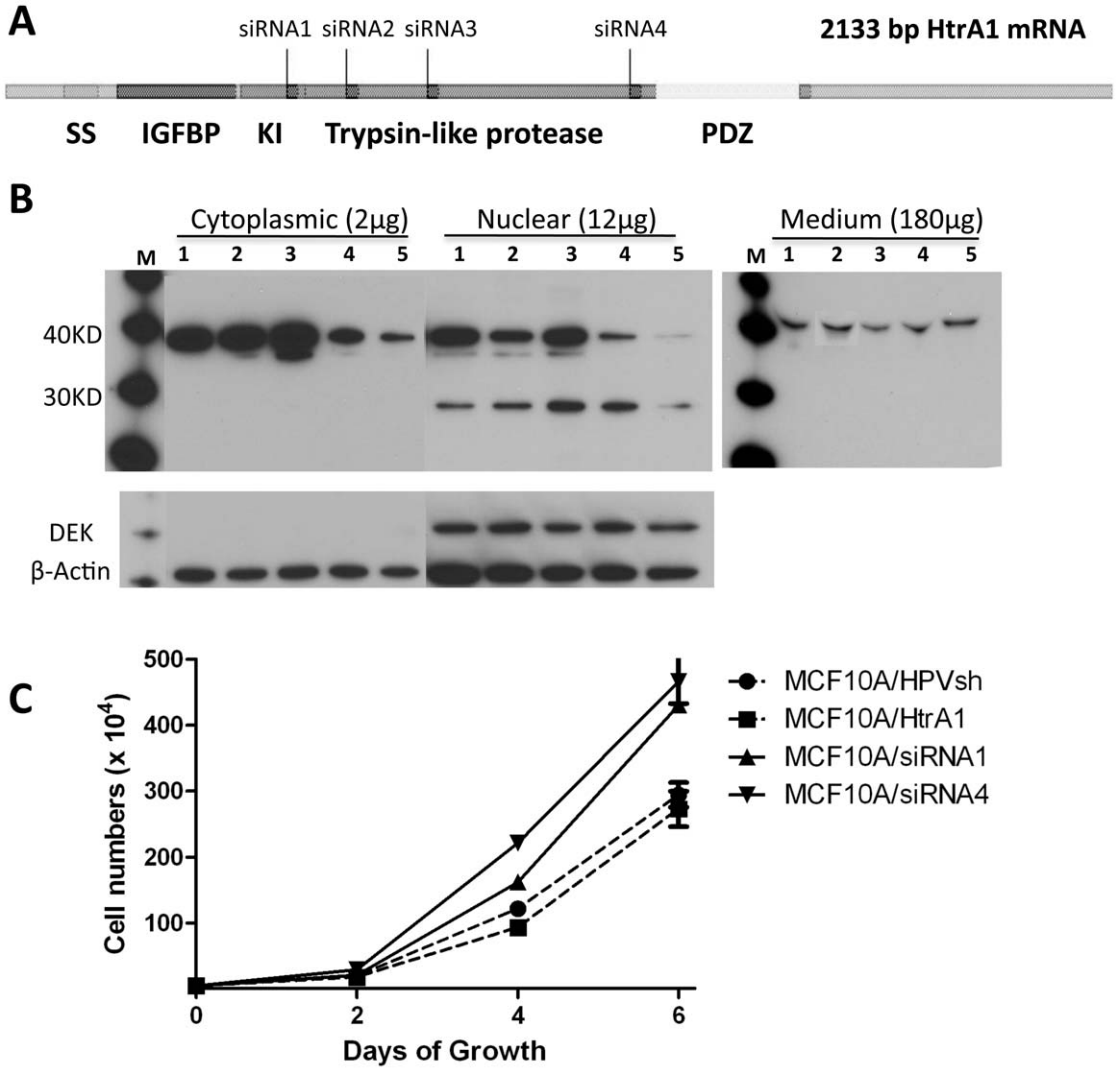
Characterization of MCF10A cell lines carrying HtrA1 siRNA and overexpression vectors. A random antisense oligonucleotide library was used to identify optimally accessible sites in HtrA1 mRNA. SiRNAs (short hairpin RNAs) were designed to target these sites, and stably transfected cell lines were developed from MCF10A cells (4 independent cell lines, designated MCF10A/siRNA1-4). A control cell line expressing an irrelevant siRNA (designated MCF10A/HPVsh) was also developed. In parallel, we also developed a cell line over-expressing HtrA1 (designated MCF10A/HtrA1). (A). Target sites empirically identified in HtrA1 mRNA. Identified domains within HtrA1 include: SS, signal sequence; IGFBP, IGF binding site; KI, Kazal Type I protease inhibitor domain; Trypsin-like protease domain; PDZ, PDZ binding domain. (B) Immunoblot analysis for HtrA1 protein. Cytoplasmic and nuclear protein fractions were prepared from the developed cell lines (as indicated), and were probed with a polyclonal antibody preparation directed against a region in the trypsin-like protease domain. As is evident, the reductions in HtrA1 protein levels were >90% compared with the various MCF10A and MCF10A/HPVsh cells. Right panel shows HtrA1 in concentrated culture medium. (C) Cells were plated and growth was measured over a 6 day period. Both of the MCF10A cell lines tested (MCF10A/siRNA1 and siRNA4) grew significantly faster than the control cells (p<0.01 at days 4 and 6). Over-expression of HtrA1 in the MCF10A/HtrA1 cells had no effect on cell growth rate.

To generate cell lines with stably down-regulated or up-regulated HtrA1 expression, we performed a library selection to identify optimally accessible target sites within HtrA1 mRNA, as previously described [37]. We selected four sites (Figure 6A), and designed short-hairpin RNAs (shRNAs) targeting these sites. We used MCF10A cells and the Flp-In system with expression vectors containing HtrA1-targeted shRNAs, and produced 4 independent cell lines with significant down-regulation of HtrA1 expression. We also created a cell line over-expressing HtrA1 by using a construct containing the 1.5 kbp full-length HtrA1 coding sequence. An MCF10A cell line carrying an shRNA targeted to human papilloma virus was used as an irrelevant control cell line (designated MCF10A/HPVsh).

The levels of HtrA1 protein expression in the over-expressing cell line (designated MCF10A/HtrA1), and the down-regulated cell lines (designated MCF10A/siRNA1-4), were evaluated using immunoblots (Figure 6B). Using an antibody raised against a 22 aa peptide within the HtrA1 protease domain, we observed doublet bands at ∼Mr 35–38,000 in the cytoplasmic extracts. In nuclear extracts, we detected an additional band at ∼Mr 29,000. In the over-expressing MCF10A/HtrA1 cell line, we observed an increase (2X) in cytoplasmic HtrA1 expression levels of the Mr 35–38,000 doublet, and a 2- to 3-fold increase in the proteolytically active Mr 29,000 nuclear form. In the various MCF10A/siRNA1-4 cell lines, we observed markedly decreased (≥90%) expression of HtrA1, relative to both the parental MCF10A and the MCF10A/HPVsh vector control cell lines (Figure 6B).

Compared to the parental MCF10A cell line, the MCF10A/HtrA1 siRNA cells appeared to be smaller, whereas the over-expressing MCF10A/HtrA1 cells appeared to be larger, and these observations were confirmed by measuring cell areas (data not shown). In cell growth experiments (Figure 6C), down-regulation of HtrA1 significantly increased cell growth rate (p<0.01 at days 4 and 6). Over-expression of HtrA1 induced a slightly slower growth rate, but this was not statistically significant.

**Figure 7:**
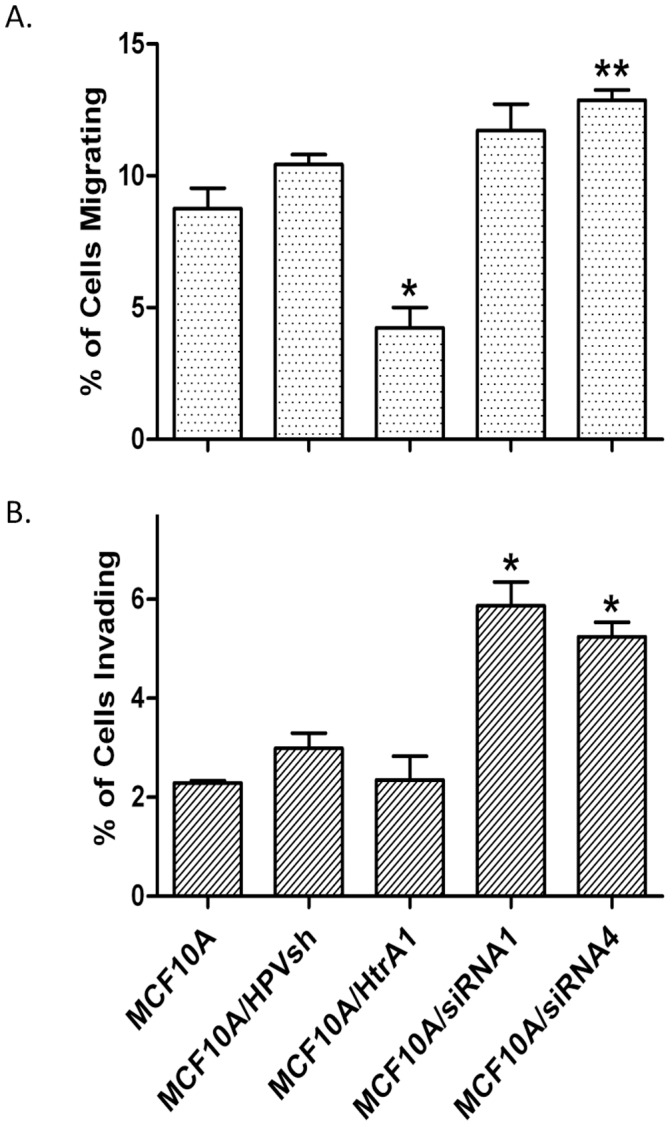
Migration and Invasion Assay. The MCF10A-derived cell lines were tested for migration and invasive capability in a transwell assay, using uncoated (migration) or basement membrane-extract coated (invasion) wells. Results are from 3 independent experiments. Panel A: Migration. The vector control cell line MCF10A/HPVsh did not differ in migration from the parental MCF10A cell line. The MCF10A/HtrA1 cell line over-expressing HtrA1 showed significantly decreased (p<0.01) migration vs. the control cell lines. However, MCF10A/siRNA4 cell line showed significantly increased migration (p<0.01), whereas the MCF10A/siRNA1 showed an increase of borderline significance. Panel B: Invasion. The MCF10A/HPVsh and MCF10A/HtrA1 cell lines did not differ in invasion capability from the parental MCF10A cell line. However, both MCF10A/siRNA1 and 4 cell lines showed significant increases (p<0.01) in invasive capability.

We next examined the migration and invasion capabilities of the various cell lines in a transwell assay. We found that one of the two MCF10A/siRNA cell lines tested showed significantly increased migration ability (*p-*value <0.01), while the MCF10A/HtrA1 over-expressing cell line showed a significant decrease in migration (*p-*value <0.01) (Figure 7A). Both the MCF10A/siRNA1 and/siRNA4 cell lines showed significantly increased invasion ability compared to the control cell line (*p-*value <0.01; see Figure 7B), while the vector control and over-expressing MCF10A/HtrA1 cell lines showed no change in invasion capability.

**Figure 8:**
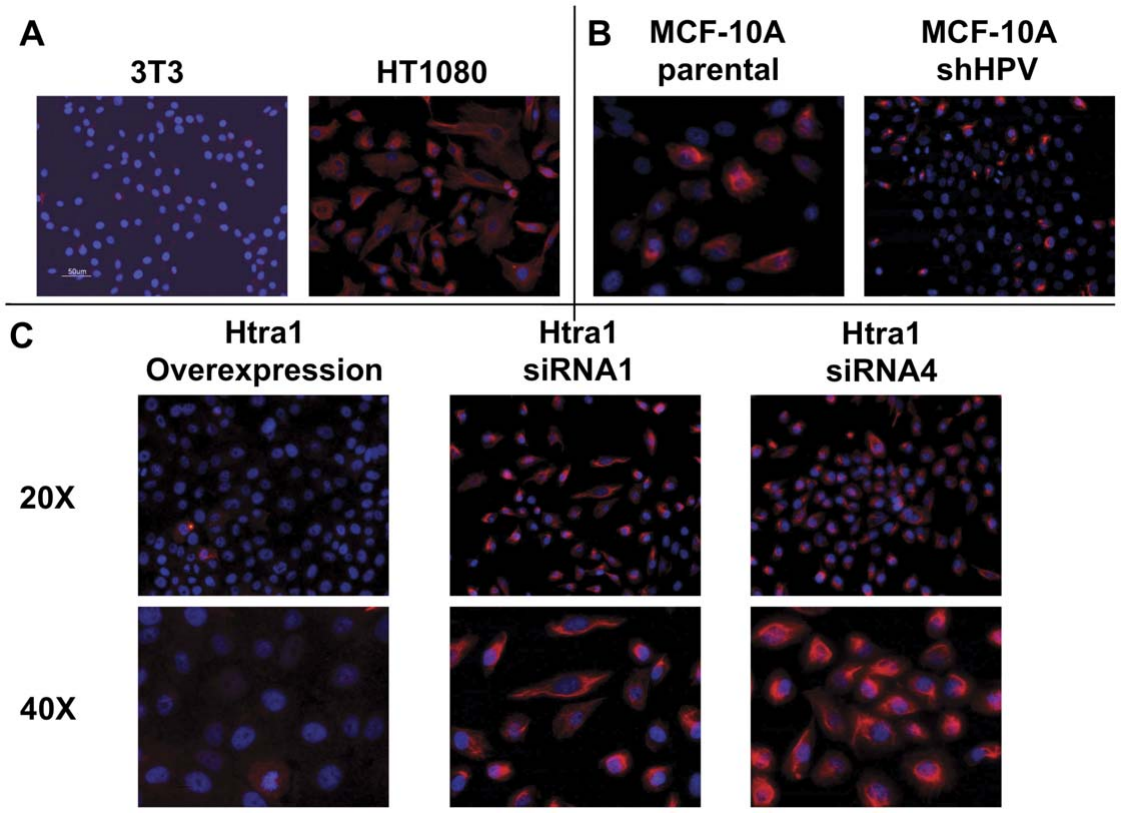
Vimentin Expression in the MCF10A-derived cell lines. IHC was performed for VIM as described (orange). Cells were also stained with DAPI to delineate nuclei (blue), and images were superimposed. Panel A: VIM staining in positive (HT1080 cell line, 40X magnification) and negative (3T3 cell line, magnification 20X) control cell lines. Panel B: VIM staining in the parental MCF10A and the MCF10A/HPVsh vector control cell lines. The MCF10A panel shows a representative high power (40X) field, in which 7 of 25 cells stain for VIM. In the MCF10A/HPVsh vector control cell line, a representative low power (20X) field is shown, with 5–10% of the cells staining for VIM. Panel C: VIM staining in the over-expressing MCF10A/HtrA1 cell line and the under-expressing MCF10A/siRNA1 and MCF10A/siRNA4 cell lines. With the MCF10A/siRNA cell lines, essentially 100% of cells show intense staining for VIM, whereas essentially all of the HtrA1 over-expressing cells do not express VIM. Fields show 20X and 40X magnifications as indicated. Controls included reactions with no primary antibody, and these were uniformly blank.

Acquiring such an increased motile phenotype is one of the typical features of the EMT. Thus, we examined expression of VIM, as a marker for mesenchymal differentiation, in the various MCF-10A modified cell lines. Interestingly, VIM staining was heterogeneous in the parental MCF10A cell line (Figure 8B), where ∼25–30% of cells in the sampled populations displayed positive staining. In this regard, breast cancer epithelial cell lines (specifically including MCF10A cells) have been shown to exhibit heterogeneous staining for CD44, CD24, and epithelial specific markers [38], which presumably correlates with the heterogeneous VIM staining in the parental control MCF10A cells. Similarly, ∼10–15% of MCF10A/HPVsh vector control cells showed positive VIM staining. In contrast to control cells, essentially all MCF10A/siRNA cells under-expressing HtrA1 showed uniformly strong, positive VIM staining, whereas the MCF10/HtrA1 over-expressing cells showed little or no VIM staining (Figure 8C). In addition, MCF10A/siRNA cell lines showed markedly decreased staining for the epithelial and myoepithelial cell biomarkers KRT5/6/18 (data not shown).

#### Effects of modulating HtrA1 levels on global gene expression profiles

**Figure 9:**
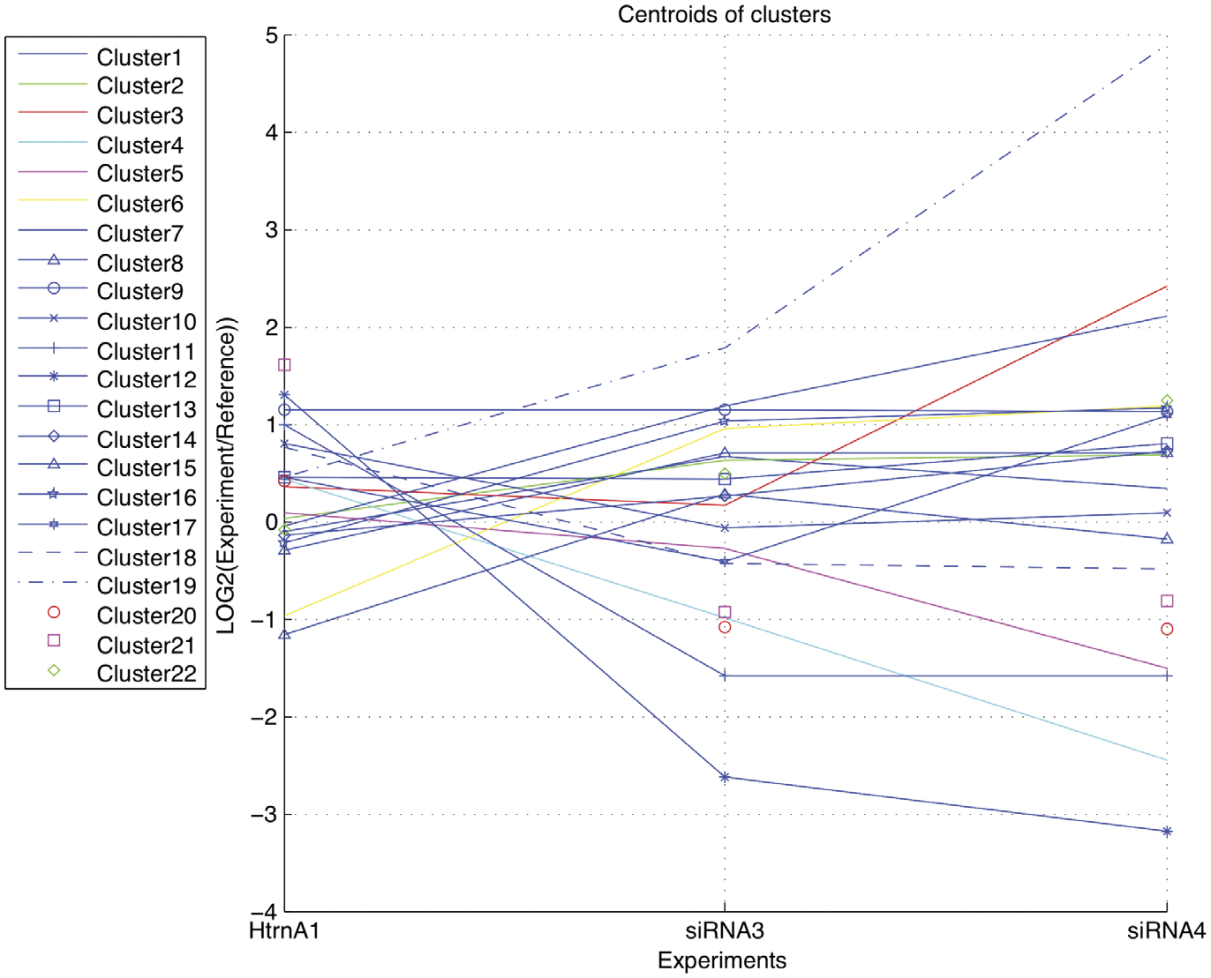
Cluster Analysis of Whole Genome Gene Expression Data. Genes showing significant changes (and a minimum of 50% change) in expression levels (at p<0.01) in MCF10A/siRNA cell lines vs. controls (3 independent experiments) were analyzed by Cluster Analysis. Gene expression profiles in MCF10A/siRNA3 and siRNA4 cell lines, and in the MCF10A/HtrA1 over-expressing cell lines, were determined vs. the control cell line. The optimal number of clusters was found to be 22, and the centroids for the various individual clusters are indicated. Clusters 19, 6, and 2 showed the greatest changes in expression levels in the MCF10A/siRNA cell lines which were inversely correlated with HtrA1 expression levels. Clusters 12, 21, and 4 showed the greatest changes in expression levels that were positively correlated with HtrA1 expression levels.

In order to understand the full range of phenotypic consequences of modulating HtrA1 levels, we examined changes in genome-wide gene expression, using the MCF10A/HtrA1 over-expressing cell line and 2 of the MCF10A/siRNA cell lines, and the Illumina Human Whole Genome Beadchip assay. We used two methods to analyze the data: gene clustering and signaling pathway analysis. First, 1402 genes were identified for cluster analysis by considering changes in expression levels in the MCF10A/siRNA cells that were greater than 50% compared to the control cell line (at p<0.01). We used the Silhouette measurement to determine the optimal number of clusters. Using this analysis, the optimal number of clusters was determined to be 22 (Silhouette width  = 0.384) (Figure 9). We detected genes whose expression was inversely correlated with HtrA1 expression level, as well as genes whose expression was positively correlated with HtrA1 expression levels. Clusters 19, 6, and 2 were the top three clusters, based on the magnitude of changes in gene expression levels, which showed expression changes inversely correlated with HtrA1 expression level. As examples, cluster 19 (4 genes) contained VIM (2 loci), cluster 6 (21 genes) included FGFR3, IGFBP2, and TNFRSF6B, and cluster 2 (281 genes) included many interesting genes such as LAMB1, RAD21, ATM, HIF1A, FGFRL1, VEGFB, VEGFC, H2AFX, MTA1, and PTPRE, PTPLA, and POLR3GL. Clusters 12, 21, and 4 were the top four clusters positively correlated with HtrA1 expression level. Cluster 12 (10 genes) included CD24, cluster 21 (23 genes) prominently included a number of histone genes, and cluster 4 (22 genes) included PRSS8, KRT15, CLDN7, and CDH1. We confirmed changes in transcript levels for many of the pertinent genes using QPCR, including VIM, CDH1, CLDN1, and ATM (not shown).

The gene expression profiling results highlighted several interesting changes. First, many EMT marker genes and EMT-related transcription factors were significantly changed when HtrA1 expression was down-regulated (Table 1). We observed substantially increased expression of mesenchymal marker genes such as VIM (9 to 34X), ECM2 (1.3 to 1.6X), and LAMB1 (1.8 to 2.2X). The direct regulators of these genes include several classes of transcription factors, which were also significantly elevated (p<0.01), including bHLH, TWIST, forkhead, and homeobox factors (data not shown). Concomitantly, down-regulation of HtrA1 significantly decreased expression of many epithelial markers, including E-Cadherin (CDH1), claudin1 (CLDN1) and claudin7 (CLDN7), and many cytokeratins (such as KRT 8), and over-expression of HtrA1 significantly decreased expression of these markers (Table 1). The TGFβ signaling pathway can be a prominent driver of the EMT [39]–[40]. However, in our experiments we did not detect any consistent inverse correlation between HtrA1 levels and the expression of TGFβ1 in the microarray results. We also did not observe any relationship between HTrA1 and TGFβ1 levels in ELISA assays from cell culture media (not shown).

**Table 1:** Selected significantly altered genes associated with EMT, ATM-DDR, and CSC pathways.

Gene Symbol	siRNA4	siRNA3	HtrA1
EMT Marker Genes			
CDH1	0.091	0.826	1.381
CLDN1	0.259	0.374	1.454
CLDN7	0.114	0.681	1.135
VIM	33.634	8.976	0.590
ECM2	1.321	1.617	0.675
LAMB1	1.795	2.188	1.074
KRT8	0.417	0.508	1.485
ATM DNA Damage Response Components			
ATM*	1.947, 1.775	1.662, 1.501	0.946, 0.992
H2AFX	1.502	1.651	1.115
H2AFY2	1.291	1.403	0.917
NASP	1.705	1.669	0.828
TP53	2.092	2.225	1.066
TP53BP1	1.254	1.240	0.923
RBBP8	1.487	1.347	0.923
AKT1*	1.348, 1.420	1.474, 1.071	0.969
POLM	1.547	1.770	0.916
RAD21	1.407	1.500	1.018
ALDH1B1	1.187	1.221	0.924
Breast Cancer Stem Cell Markers			
CD24*	0.083, 0.062	0.175, 0.190	2.515, 2.129
CD44	2.308	1.877	1.256
ALDH1L1	3.572	2.647	0.869
Angiogenesis and HIF Signaling Pathways			
VHL	1.321	1.361	0.728
HIF1A*	1.850, 1.739	1.746, 1.514	1.091, 1.033
VEGFB	1.630	1.472	0.946
VEGFC	1.665	1.563	1.137
FGFR4	1.576	1.788	0.852
FGFR3	2.597	1.435	0.577
FGFBP1	2.400	1.500	0.718
EGFR	1.381	1.326	0.988

Values represent fold change relative to MCF10A vector control values. All values are significant at p<0.01. * Genes with 2 independent loci measured.

Cancer stem cells (CSC) can self-renew and differentiate to recapitulate the cellular heterogeneity of the original tumor [41], and Al-Hajj et al. have isolated CD44^+^, CD24^−/low^ breast cancer initiating cells with CSC-like properties [42]. Surprisingly, we observed an ∼90% decrease of CD24 gene expression in MCF10A/siRNA cell lines, with an average 100% increase in CD44 expression (Table 1). We also detected a 2.6 to 3.6X increased expression of ALDH1L1 in the same HtrA1 down-regulated cells (Table 1), accompanied by parallel changes in a number of additional ALDH1 transcripts. Whether a subpopulation of putative CD24−/CD44+/ALDH1+ cells truly exist in the MCF10A/siRNA cells is unclear, but our results could point to an additional role for HtrA1 in breast carcinogenesis.

The MCF10A/siRNA cell lines showed significant transcriptional up-regulation of many components in the nuclear protein kinase ataxia telangiectasis mutated (ATM)-initiated DNA damage response network; ATM is a major sensor of DNA damage. In addition to ATM, expression of signaling mediators, such as 53BP1, MDC1 and MCPH1, and downstream targets, such as p53, H2AFX, H2AFY2, NASP, and RAD21, were all increased when HtrA1 levels were decreased (Table 1). Levels of many of the same genes showed contrasting decreases in HtrA1-overexpressing cells (Table 1). With regard to DNA metabolism, we also observed significant (p<0.01) coordinated transcriptional up-regulation of a number of DNA polymerases, including PolM, PolE2, PolD3, and PolS, suggesting that both replicative and repair DNA machinery was affected by HtrA1 down-regulation.

Finally, pathways important in cancer progression were affected by HtrA1 expression levels. For example, factors within the angiogenesis and HIF pathways, such as HIF1A, VHL, FGF family members, AKT1, and VEGFB/C genes, all showed major increases in the HtrA1 down-regulated MCF10A/siRNA cell lines and decreases in the MCF10A/HtrA1 over-expressing cell line (Table 1). Each of the MCM2-7 genes showed coordinated down-regulation, which is of interest because these MCM proteins are negative regulators of HIF1 [43] and exposure to hypoxia leads to their down-regulation. We also observed that AKT was phosphorylated (at Ser473) in the MCF10A/siRNA cell lines using immunoblot analysis (not shown); this site is involved in control of AKT activity [44], and is of further interest because it predicts chemoresponsiveness to paclitaxel in breast cancer [45].

### Functional Characterization of the DDR in Response to Altered HtrA1 Expression

**Figure 10:**
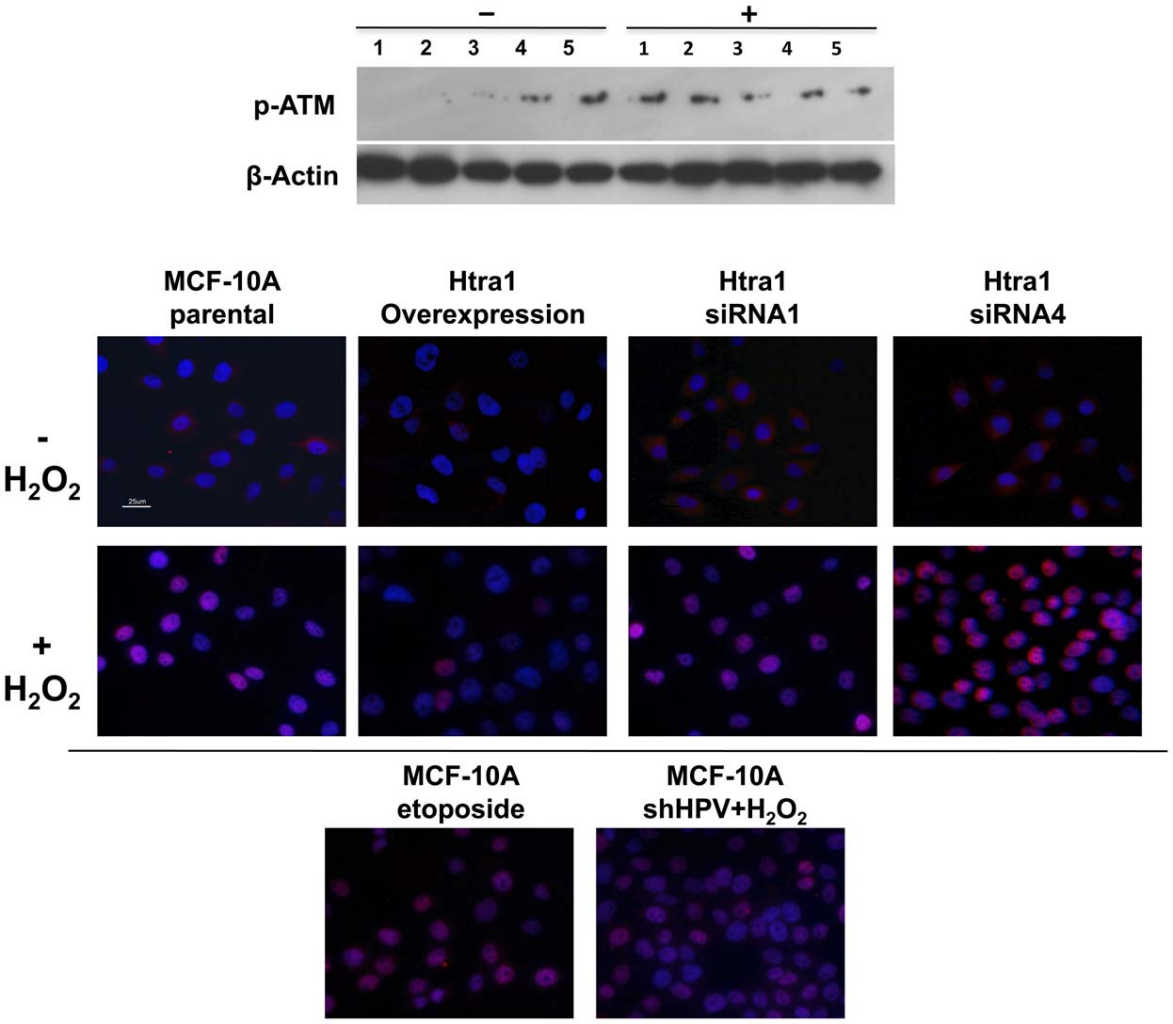
Examination of MCF10A-derived cell lines for p-ATM and formation of γH2AX foci. Upper Panel. Immunoblot analyses for phosphorylated ATM were performed as described, specifically examining the Ser1981 site. The Upper Panel shows phosphorylated ATM (with β-actin as a loading control). “+” indicates H_2_O_2_ treatment with 100 µM H_2_O_2_ for 2 h to induce oxidative stress, vs. “-“ for no treatment. Numbers indicate cell lines as follows: 1 = MCF10A, 2 = MCF10A/HtrA1, 3 = MCF10A/HPVsh, 4 = MCF10A/siRNA1, and 5 = MCF10A/siRNA4. Phosphorylated ATM (pATM, at Ser1981) is observed in the absence of any treatment in both MCF10A/siRNA cell lines examined, whereas no pATM is observed in the MCF10A and MCF10A/HPVsh control cell lines, or the over-expressing MCF10A/HtrA1 cell line. **Lower Panels.** Cells were either untreated or treated with 200 µM H_2_O_2_ for 1 h, and then IHC analysis was performed, with staining for γH2AX foci using an antibody specific for phosphorylated Ser139 as described. Over-expression of HtrA1 effectively blocked formation of γH2AX foci following acute treatment with H_2_O_2_, whereas foci formation was increased in MCF10A/siRNA1 (generally by ∼50%) and/siRNA4 cells, dramatically so in the latter. Etoposide treatment was used as a positive control with MCF10A cells. Foci formation is unchanged in the MCF10A/HPVsh vector control cell line. Results are from a representative experiment, which was repeated once with analogous results.

We examined whether the transcriptional up-regulation of ATM reflected activation of the functional pathway, via immunoblot analyses for phosphorylation of ATM at Ser1981. In the absence of exogenously induced oxidative stress, phosphorylated ATM bands were observed in both MCF10A/siRNA1 and/siRNA4 cell lines examined, suggesting that ATM was activated. Phosphorylated ATM was not observed in control cells (MCF10A and MCF10A/HPVsh) or in the MCF10A/HtrA1 over-expressing cell line (Figure 10, upper panel) in the absence of induced stress.

In turn, activated ATM can phosphorylate Ser139 of H2AX (forming γH2AX) at the site of a double strand DNA break (DSB) [46]. Therefore, to test whether an ATM-initiated DDR is functionally activated in the MCF10A/siRNA cells, we treated parental MCF10A cells, HtrA1 over-expressing MCF10A/HtrA1 cells, and the MCF10A/siRNA4 and/siRNA1 cells (and appropriate controls) with H_2_O_2_ (200 µM, 1 hr), to induce oxidative stress. As a positive control for formation of DSBs, the MCF10A parental cell line was also treated with 25 µg/ml etoposide. Stained γH2AX foci were observed in the H_2_O_2_-treated MCF10A groups, but not in the untreated MCF10A cells (Figure 10, lower panels). γH2AX staining intensity was significantly decreased in the MCF10A/HtrA1 over-expressing cell line, and was significantly increased in the MCF10A/siRNA4 and/siRNA1 cell lines (Figure 10).

#### Decreased HtrA1 expression results in down-regulation of microRNA 200 family members

Given the important roles of miRs in cellular regulation, we also performed a genome-wide analysis of miR profiles in the various MCF10A cell lines using the Illumina v2 MicroRNA Beadchip Assay, containing 1146 miR genes. An unsupervised analysis of miRs showed that 291 miRs showed differential expression in the analyses, using p<0.05 as a screening criterion. By focusing on changes in expression of at least 40%, with a p-value <0.05, the number of differentially expressed miRs was reduced to 140 (MCF10A/siRNA1,/siRNA2,/siRNA3, and/siRNA4 cell lines had 53, 16, 32, and 39 significantly changed miRs, respectively), vs. the control MCF10A/HPVsh cell line.

Cluster analysis showed that the optimal number of clusters for the p<0.05 results was 22. Cluster 3, for example, contained miR-429, 200a, 200a*, 200b, 200b*, and 376c, while closely related Clusters 6 and 8 contained miR-200c* and 141, respectively.

Imposing a False Discovery Rate (FDR) of <0.05 reduced the number of differentially expressed miRs to 41 across the MCF10A/siRNA cell lines. Using the FDR correction, the optimal number of clusters was 19. Cluster 1 contained miR-200a, 429, 200b, and 200a*, while Cluster 6 contained 200c*. With an exceedingly stringent Sidak correction, only 4 differentially regulated miRs were identified; miR-200a, 345, 376c, and 888. miR345 is a methylation-sensitive miR (down-regulated) which is involved in cell proliferation and invasion in colorectal cancer [47], while miR-376c (here up-regulated) enhances ovarian cancer cell survival and has been implicated in chemoresistance [48].

Considering all analyses, the miR-200 family members (miR-429, 200a(a*), 200b(b*), 200c(c*), and miR-141) were consistently identified as differentially regulated in response to altered HtrA1 expression (Table 2). The miR-200 family members showed highly significant decreases in expression with ranged from ∼40% to more than 90%. One other miR showing a major change was miR-34c-3p. miR-34c-3p has been found to produce decreases in anchorage-independent growth, migration, and invasion in siHa cells [49], and miR-34 has also been found to be down-regulated in prostate cancer [50], where it otherwise exhibits tumor suppressor properties. Stinson et al. have recently reported that miR-221/222 promotes the EMT in breast cancer by targeting TRPS1 [51]. In contrast, here we observed increased expression of miR-221/222 with overexpression of HtrA1 (221, 221*, 222, and 222* levels were 1.25, 1.65, 1.38, and 1.50 vs. control, respectively) and unchanged or slightly decreased expression in the MCF10A/siRNA1-4 cell lines (221, 221*, 222, and 222* were 0.82±0.11, 1.04±0.33, 0.89±0.08, and 1.47±0.45 vs. control, respectively).

**Table 2:** Effects of Altered HtrA1 Expression Levels on the miR-200 Cluster.

miR	HtrA1		siRNA1-4
Effects on Expression of miR-200 Family Members			
429	1.35±0.10	0.34±0.30	
200a	1.44±0.16		0.46±0.31
200a*	1.25±0.25		0.61±0.27
200b	1.32±0.03		0.41±0.21
200b*	1.48±0.19		0.52±0.24
200c	1.21±0.13		0.77±0.06
200c*	1.18±0.20		0.68±0.30
141	1.14±0.02		0.67±0.12

Two independent analyses were performed, which included the MCF10A vector controls, all 4 different MCF10A/siRNA1-4 cell lines, and the over-expressing MCF10A/HtrA1 cell line. Values for the various cell lines were normalized to the vector controls. We initially focused on miRs showing significant changes (p<0.05) in the MCF10A/siRNA cell lines, of at least 40% magnitude. This initial screening identified a small group of miRs, most of which were members of the miR-200 family. Values for these miRs were then extracted from the data, and all were found to be statistically significantly different at p<0.05 or greater.

## Discussion

Here, using a high quality affinity-purified antibody, we demonstrated that HtrA1 is strongly expressed in normal ductal epithelium in human breast tissue specimens. Expression patterns included strong epithelial staining (both cytoplasmic + nuclear, or prominently nuclear), as well as a pattern of intense HtrA1 staining of the basement membrane surrounding ducts (also including blood vessels; Figure 1, N panels). These diverse staining patterns were characteristically observed focally throughout the same tissue specimens, and presumably reflect the diversity of microenvironmental influences. In marked contrast, HtrA1 expression was greatly reduced or lost entirely in DCIS and invasive carcinomas (Figure 1, CA panels). We are currently expanding our IHC studies, using a multiplexed approach to co-localize HtrA1, VIM, and 4-hydroxynonenal, as well as additional markers of oxidative stress.

In concordance, HtrA1 expression was significantly reduced in all of the breast cancer cell lines examined, compared with their non-tumorigenic counterparts. HtrA1 gene expression silencing was due to epigenetic modifications, including at least promoter DNA hypermethylation, histone deacetylation, and translation inhibition. These experimental findings provide empirical support for the prediction that HtrA1 would be epigenetically regulated in breast cancer cell lines [52].

For mechanistic studies, we created MCF-10A cell lines which stably under- or over-expressed HtrA1. We found that substantially decreased expression of HtrA1 stimulated cell growth and triggered the EMT. Evidence of EMT included acquisition of mesenchymal attributes, such as expression of various markers (VIM, etc.) and functional properties (increased invasion). Global gene expression studies showed down-regulation of numerous epithelial markers (CDH1 and CLDNs) and major decreases in the miR200 family, known regulators of the EMT. In addition to the EMT changes, we observed alterations in the expression profiles of many CSC-associated genes and ATM DDR pathway components in response to changes in HtrA1 levels, potentially providing a link to HtrA1’s puzzling role in response to chemotherapeutics. Functionally, we demonstrated activation of the DNA damage sensor, ATM, in cells with decreased HtrA1 levels, and we found that HtrA1 expression levels were inversely correlated with formation of γH2AX foci in response to an acute oxidative insult. Interestingly, we have also made a number of attempts to over-express HtrA1 in MCF7 cells. In all attempts, no over-expressing clones could be obtained, so “re-expression” of HtrA1 in this context may produce cell death.

Proteases play essential roles in multiple biological processes. Beyond their functions in protein catabolism, proteases can selectively cleave substrates and thus influence cell behavior, survival, and death [53]. For many years, proteases (especially extracellular) have been implicated in tumor progression, with the probably overly simplistic assumption that they can degrade extracellular matrix thus facilitating cell migration and invasion (see [54]). However, this assumption has not translated into clinical utility; in recent clinic trial studies, treatment of patients with broad-range metalloproteinase inhibitors showed no effects, or even produced an acceleration of tumor growth [55], [56]. This finding suggests that some extracellular proteases might actually have anti-tumor properties. For example, the serine proteases PRSS3 (also known as trypsinogen IV), PRSS8 (prostasin), and PRSS21 (testisin) were categorized as tumor-protective proteases in the human degradome [57]. We suggest that HtrA1 may represent another tumor suppressor in this group.

The EMT is fundamental during embryonic development, and has a parallel role in tumorigenesis [58], [59]. More importantly, EMT has been found to contribute to tumor invasion, metastasis, and acquisition of therapeutic resistance. Therefore, targeting EMT-associated processes is a promising avenue in chemotherapies. We demonstrate that HtrA1 expression levels are inversely correlated with motility and invasion (Figure 7), and acquisition of increased motility is one of the typical features of EMT. Down-regulation of HtrA1 significantly decreased many “epithelial” genes, including E-Cadherin, claudins, and cytokeratins, whereas mesenchymal marker genes such as vimentin, ECM2, and LAMB1 showed increased expression in the MCF10A/siRNA cell lines (Table 1). The direct regulators of these genes include several classes of transcription factors, many of which were highly expressed in the MCF10A/siRNA cell lines. Hypoxia is another event that can promote the EMT [43], [60], [61], [62], [63], [64], via mechanisms involving HIF-1a. In fact, HIF-1a has been found to induce genetic alterations by suppressing DNA repair [60], [61], and short-term hypoxia induces a reversible EMT that requires the transcription factor Twist1 [60]. Many components within the angiogenesis and HIF pathways were affected by HtrA1 down-regulation (Table 1). Finally, the EMT has been associated with acquisition of a cancer stem cell (CSC)-like phenotype [34]. In our microarray analysis data, we observed that decreased expression of HtrA1 was associated with acquisition of the breast CSC phenotype CD24−/CD44+/ALDH1+ (Table 1). This may relate to the phenotypic diversity exhibited by breast epithelial cell lines [38] which we also observed.

The central finding from our miR analyses was that the entire miR-200 family was down-regulated in our analyses of MCF10A/siRNA cell lines (Table 2). Importantly, the miR-200 transcriptional cluster has recently been found to be epigenetically controlled by methylation of the miR-200 promoter [65], [66], [67], so this may represent a mechanism underlying our results. This family of miRs may directly regulate EMT transcription factors, such as ZEB1, ZEB2 [68], or may act on their target genes. For example, PTPN12 is one of their known targets [32]. Recently, Lliopoulos et al. showed a decrease in the ratio of Akt1 versus Akt2 in cells induced the down-regulation of miR-200, and promoted the EMT and a CSC-like phenotype [33].

The TGFβ signaling pathway can be a prominent driver of the EMT [39]–[40]. The pleiotropic nature of TGFβ indicates that it has a complex role in tumor progression. On the one hand, TGFβ signaling pathways are often lost in hepatic, pancreatic, gastric and colorectal carcinomas [69]. On the other hand, in several cancer types high levels of TGFβ in patient serum are associated with poor prognosis [70], [71], [72]. In an in vitro experiment, Oka et al. showed HtrA1 bound to a broad range of TGFβ family proteins, including Bmp4, Gdf5, TGFβs and activin in a GST-pulldown assay in mouse myoblast C2C12 cells [73]. However, in our experiments we did not detect any consistent inverse correlation between HtrA1 levels and the expression of TGFβ1 in the microarray results. These data suggest that HtrA1 might act downstream of TGFβ, or perhaps in a parallel pathway regulating the EMT.

Normal cellular stress response is an important barrier to carcinogenesis. As one type of cellular stress, oxidative damage can arise from overproduction of ROS and deficient antioxidant and/or DNA repair systems [74]. ROS-induced DNA damage can result in base modification, deoxyribose modification, DNA cross-linking, and single- and double-strand breaks. If such damage is not repaired before replication, replication errors, mutations, cell death, and even genomic instability may occur [75]. A number of interesting aspects regarding DDR pathways are evident in our study. First, ATM was activated (phosphorylated) following down-regulation of HtrA1 expression, even in the absence of exogenous stress (Figure 10). Recently, the ATM protein was identified as a cellular redox sensor, in addition to its well-defined role in DNA repair signaling [76]. Direct oxidation of the ATM protein resulted in ATM activation, in the absence of DNA strand breaks. Therefore, our observed ATM activation in untreated MCF10A/siRNA cells may indicate that loss of HtrA1 results in increased endogenous intracellular ROS levels. After acute oxidative stress, we observed that MCF10A/siRNA cells displayed an increased number of γH2AX foci, relative to control cells, further suggesting that loss of HtrA1 correlates with higher levels of DNA damage. In contrast, high levels of HtrA1 may protect cells against ROS-induced DNA damage. We speculate that the absence of HtrA1 may be associated with genomic instability, in which case it becomes important to investigate HtrA1’s role in cellular redox regulation and responses to oxidative stress.

Second, we observed the transcriptional up-regulation of many mediators in the ATM-arm of the DDR pathway [77], [78], [79]; the ATR-arm of the pathway did not appear to be affected (data not shown). However, we did see up-regulation of PARP1, so single-strand break levels may also be increased following down-regulation of HtrA1.

Third, down-regulation of HtrA1 resulted in a vigorous increase in hypoxia-response genes (Table 1). Chronic hypoxia has been found to decrease synthesis of homologous recombination proteins, which could result in error-prone DNA repair with significant ramifications for genomic instability and chemotherapeutic responses [80].

It would be of interest investigate HtrA1’s role in chronic oxidative stress, to determine whether there is therapeutic value in trying to restore its activity in breast cancer cells, since it is otherwise epigenetically silenced. Chien and co-workers [11] showed that HtrA1 expression enhanced sensitivity to cisplatin and paclitaxel, whereas down-regulation attenuated cytotoxicity. Folgueira et al. identified HtrA1 as one of a cohort of only 3 genes (HtrA1, MTSS1, CLPTM1) that could distinguish doxorubicin-responsive from non-responsive tumors in 95% of the samples [19]. The anti-tumor mechanism of doxorubicin involves both inhibition of topoisomerase and DNA synthesis [81] and generation of ROS through redox-activation [82]. In this regard, it is of interest that Creighton et al. [83] found that breast cancer cells surviving after chemotherapy showed changes indicative of the EMT, although this is undoubtedly a multifactorial event.

Breast cancer is not a single disease, but rather is comprised of diverse subtypes with different molecular features, which may influence clinic outcomes. While many advances in therapeutic approaches have been made, much improvement is still needed; many patients receiving systematic therapy for breast cancer either do not need it or will not benefit from it. Improved biomarkers are required to accurately determine whether therapy will be appropriate, and HtrA1 may serve as one such valuable early biomarker.

## Materials and Methods

### Cell Lines

The MCF10A, MCF12A, MCF7, MDA-MB-231, MDA-MB-468, NM2C5, and M4A4 cell lines were obtained from ATCC (www.atcc.org).

### Derivation of MCF10A-based Cell Lines

The Flp-In system (Invitrogen) was used to create stably transfected MCF10A-derived cell lines using a two–step sequential procedure. First, pFRT/lacZeo plasmid DNA was purified and linearized with ApaI endonuclease and then transfected into the parental MCF10A cell line. This construct encodes a Flp-recombination site. Zeocin antibiotic (80 µg/ml) was used for selection of stable integrants (for ∼30 days). MCF10A/Flp positive clones were verified by β-gal staining and screened by Southern analysis to identify single-copy clones for the next transfection. Second, the pcDNA5/FRT expression vectors were produced, which contained either: a) the HtrA1 full-length coding sequence, which was driven by human CMV promoter; or b) HtrA1-targeted siRNAs, which were driven by two opposing Pol III promoters, H1 and U6 (see [84]). These were co-transfected with pOG44, a plasmid encoding the Flp recombinase, into MCF10A/Flp cell line. Hygromycin antibiotic (30–40 µg/ml, for ∼30 days) was used to select cells stably overexpressing HtrA1 (denoted MCF10A/HtrA1) or stably expressing the HtrA1-targeted siRNAs (4 separate siRNAs were used to produce 4 different siRNA-expressing cell lines, denoted MCF10A/siRNA1-4). Target sites for the siRNAs were identified by library selection of accessible sites as previously described [37]. Positive clones were checked for loss of β-gal activity, and HtrA1 expression levels were determined.

The full-length mRNA for the coding region of HtrA1 was 113–1555 bp (the HtrA1 sequence, 2133 bp, NM_002775 in NCBI GenBank, is 2133 bp).

The sequences used for siRNAs1-4 were:

*siRNA1: GATCTAAAAAGCCGCCGGTCATCGTCCTGCATT (544–564 bp).*

*CTAGAATGCAGGACGATGACCGGCGGCTTTTTA.*

*siRNA2: GATCTAAAAACCGTGGTTCATATCGAATTGTTT (659–679 bp).*

*CTAGAAACAATTCGATATGAACCACGGTTTTTA.*

*siRNA3: GATCTAAAAAGGTGCCACTTACGAAGCCAAATT (819–839 bp).*

*CTAGAATTTGGCTTCGTAAGTGGCACCTTTTTA.*

*siRNA4: GATCTAAAAACACGGAGTCCCATGACCGACATT (1204–1224 bp).*

*CTAGAATGTCGGTCATGGGACTCCGTGTTTTTA.*

#### Cell growth studies

To monitor the cell growth rate in the MCF10A/siRNA and MCF10A/HtrA1 transfected cell lines, 5×10^4^ cells for each cell line were plated in 10 cm plates on day 0. Cells were continuously cultured for 6 days in DMEM/F12 medium +5% Horse serum, 20 ng/ml human epidermal growth factor, 0.01 mg/ml bovine insulin, 100 ng/ml cholera toxin, 500 ng/ml hydrocortisone, 95% and 30 µg/ml Hygromycin selection antibiotic, with culture medium replaced every 2 days. Cells were counted on day 2, day 4 and day 6, with triplicate plates for each count.

For analysis of cell size, on day 4 areas of 50 randomly-selected cells for each cell line were measured (blindly), using the ImageJ program and statistics available from http://imagej.nih.gov.

#### QPCR for HtrA1

Total RNA from MCF10A, MCF12A, MCF7, MDA-MB-231, MDA-MB-468, NM2C5, and M4A4 cells (obtained from ATCC) were extracted with Qiagen RNeasy mini kit (cat. #74904). Quantitative real-time PCR was performed as previously reported [85] with the QuantiTect Probe RT-PCR kit (Qiagen, Cat. # 204443) using a Stratagene Mx4000 QPCR systems (Agilent Technologies). All quantification data were normalized to Tata-box binding protein (TBP), which acts as an internal control. The following primers and probe were used for human HtrA1, 5′-TTGTTTCGCAAGCTTCCGTT-3′ (forward), 5′-ACGTGGGCATTTGTCACGA T-3′ (reverse), 5′-FAM-TCTAAACGAGAGGTGCCGGTGGCTAGT-BHQ-3′ (probe); for human TBP, 5′-CACGGCACTGATTTTCAGTTCT-3′ (forward), 5′-TTCTTGCTGCCAGTCTGGACT-3′ (reverse), 5′-HEX-TGTGCACAGGAGCCAAGAGTGAAGA-BHQ-3′ (probe). Data were analyzed using the manufacturer’s supplied software.

#### Northern blotting

Total RNA for each cell line was extracted with Qiagen RNeasy mini kit (cat. #74904). For Northern analysis, we used NorthernMax-Gly kits from Ambion (#AM1946). 15 µg of RNA for each sample was electrophoresed in a 1% agarose denaturing gel. RNAs were transferred to MAGNAgraph nylon membranes (Osmonics, #NJ0HYA0010). Blots were probed for HtrA1, using a 1.5kb full-length cDNA probe. A 2.1kb β-actin cDNA probe was used to document loading. Both probes were labeled with DECAprime II kit (Ambion, #AM1456). The blot was exposed to X-ray film and developed 24 h later.

#### Analysis of DNA methylation in the HtrA1 promoter region

The promoter sequence for the human HtrA1 gene was obtained from the UCSC Genome Bioinformatics website (www.genome.ucsc.edu). Possible transcription factor binding sites were examined in the 2000 bp promoter sequence before the transcription start site using the TRANSFAC database (www.gene-regulation.com/databases.html).

EpiTech Bisulfite kits from QIAGEN (cat. # 59104) was used for complete bisulfite conversion and cleanup of DNA for methylation analysis. Briefly, 1 µg genomic DNA was incubated with sodium bisulfite buffer in a thermocycler condition for 5 hr. After the incubation, converted DNAs were cleaned up with the kit. The cleanup products were directly used for PCR and sequencing.

For amplification, the F1 and R1 is the primer pair designed on MethPrimer (www.urogene.org/methprimer) was used. F1 is the forward primer 5′-TTTATTATTTTATTGTGGGTTTGGG, R1 is the reverse primer, 5′-AATAAAACTTTACAAAAAAACCCTAC, which amplifies the −561 to −266bp promoter region. Regions were sequenced 2 independent times. One used sequencing the PCR product directly, and the other time the products were cloned into TOPO vector, and the plasmid sample was then sequenced.

#### Effects of decitabine and trichostatin A on HtrA1 expression

MDA-MB-231, MDA-MB-468, MCF7, M4A4 cells were examined in these experiments. Decitabine (DEC) was from Tocris (Cat. No. 2624), and trichostatin A (TSA) was from Sigma (T8552). 5×10^5^ cells were plated in 6-cm plates for individual treatment, one day prior to treatment. For the experiment, DMSO, DEC (5µM), TSA (300nM) or DEC + TSA combination was added to medium and cells were incubated for 72 h. Total RNA was then extracted as above, and samples were used for QPCR analysis.

### Immunoblot Analyses

1. Characterization for HtrA1 expression level. Cells were extracted with NE-PER nuclear and cytoplasmic extraction reagents (Thermo Scientific #78833). 2 µg of cytoplasmic, 12 µg of nuclear, and 180 µg protein from cell culture medium were separated by electrophoresis using 10%SDS-PAGE. Proteins were transferred to Immobilon-FL PVDF membranes (Millipore, #IPFL00010), and membranes were blocked with 6% milk in TBST for 1.5 hr at RT. Primary antibody was generally a rabbit polyclonal anti-human HtrA1 (Imgenex, IMX-6518A); it was used at 1∶5000 dilution, and incubation was overnight at 4 C. Secondary antibody was anti-rabbit IgG, HRP-linked (Cell Signaling Technology, #7074). Where indicated, primary antibody was a mouse monoclonal anti-human HtrA1 (R&D Systems, #MAB2916, raised against HtrA1aa23-480). It was used at 1∶500 dilution, with anti-mouse secondary antibody.
2. As loading control, β-actin mouse monoclonal antibody from Santa Cruz (#SC-47778) was used, and for assessing the nuclear/cytoplasmic fractionation, a rabbit polyclonal anti-DEK antibody was used (Aviva Systems Biology, #P100637; DEK is a nuclear protein).
3. Phosphorylation of DNA damage response components. The same amount of cells for each cell line was plated (2×10^4^ cells/cm^2^) the day before experiment. The next day, cells were treated with 0 or 100 µM of H_2_O_2_ in medium for 2 hours. Then whole cell lysates were harvested and analyzed with mouse monoclonal anti-phospho-ATM (ser^1981^) (Upstate, #05-740). As loading control, a β-actin mouse monoclonal antibody from Santa Cruz was used (#SC-47778). Secondary antibodies were from Cell Signaling Technology (anti-mouse IgG, HRP-linked, #7076; anti-rabbit IgG, HRP-linked, #7074).

#### Immunohistochemical staining of human breast tissues

The use of tissues in this study was authorized by the Institutional Review Board. Human breast tissue from mastectomies and reduction mammoplasties were fixed in 10% neutral buffered formalin for 12–24 h and embedded in paraffin. At least 3 specimens of normal, DCIS, and invasive carcinomas were examined on multiple occasions. Formalin-fixed, paraffin-embedded (FFPE) tissues were sectioned at 5 µm, transferred onto Fisher SuperfrostPlus slides, deparaffinized through a graded alcohol series, rehydrated and then subjected to antigen retrieval using Vector Antigen Unmasking Solution, pH 6.0 (cat.# H-3300) for 1 h at 80°C. Endogenous peroxidase was inhibited with 0.3% H_2_O_2_ for 30 min at room temperature. The sections were incubated in a humidity chamber overnight at 4°C with one of 3 antibodies against HtrA1∶1) Sigma “Prestige” (cat. # HPA036655), an affinity-purified rabbit polyclonal, at a dilution 1∶50. This antibody yielded the best results; 2) Abcam (cat. # ab38610), an affinity-purified rabbit polyclonal IgG, at a dilution 1∶50; and 3) IMGENEX cat. # IMX5136 polyclonal rabbit antisera. Immunocytochemistry was completed using Vector Immpress anti-rabbit kits (cat# MP-7401) with Vector Immpact DAB (cat. #SK-4105 ) as chromagen. Where indicated, Vector Methyl Green was used as a counterstain; this was generally necessary in the DCIS and invasive cancer specimens, since HtrA1 staining was greatly reduced or lost. Negative controls routinely included adjacent sections from each sample that were incubated with 2.5% normal horse serum provided without the primary antibody.

#### Cellular immunofluorescence staining

Cells were plated at 2–3×10^4^ cells/cm^2^ on the chamber slides the day before staining. On the experiment day, cells were rinsed with PBS first, then fixed in 10% neutral buffered formalin for 20 min at RT. After rinsing with PBS, cells were permeablized in PBS/0.2%Triton-X for 10 min at RT and then blocked with 2.5% secondary species serum in PBS/0.1% Triton-X for 1hr at RT. Primary antibody was diluted in the same blocking buffer and incubated with samples at 4^o^C overnight. On the following day, cells were washed with PBS/0.2%Triton-X 3X for 3min, and then incubated with secondary antibody for 1 hr at RT. They were washed again with PBS/0.2%Triton-X 3X for 3min. After the final wash, DAPI was used to counterstain cells for 5min and they were then mounted for microscopy. Antibodies used were:

1. Mouse monoclonal to Vimentin (V9) (NeoMarkers)
2. Donkey anti-mouse secondary antibody (Cy3)
3. Mouse monoclonal to Cytokeratin 5+6+18 (Abcam, #ab49289)
4. Rabbit polyclonal secondary antibody to mouse IgG-H&L (FITC) (Abcam, #ab97045)

#### Analysis of phosphorylated γH2AX foci

Cells were plated at 4×10^4^ cells/cm^2^ on the chamber slides the day before staining. 24 hours later, cells were treated with hydrogen peroxide at 200 µM for 1 hr. This time was chosen after time-course experiments. Where indicated, etoposide (at 25 µg/ml) was applied to serve as a positive control for formation of DNA double-strand break. After the treatment, cells were fixed with 10% neutral buffered formalin for 20 min at RT. After rinsing with PBS, cells were permeablized in PBS/0.2%Triton-X for 10 min at RT and then treated with 2.5% goat serum in PBS/0.1% Triton-X for 1hr at RT. Then biotin-conjugated anti-phospho-H2AX (Millipore, #16-193, which is specific for phosphoserine 139) antibody was diluted in the same blocking buffer and incubated with samples at 4^o^C overnight. On the following day, cells were washed with PBS/0.2%Triton-X 3X for 3 min, and then incubated with tetramethylrhodamine-conjugated streptavidin antibody (Molecular Probes #S870) for 30 min at RT, followed by washing with PBS/0.2%Triton-X 3X for 3min.

#### Cell migration and invasion assays

96 well HTS transwell permeable supports with 8 µm pores were obtained from Corning (Cat. No. 3374) and used for migration and invasion assays. 5x Basement Membrane Extract (BME) coating solution was obtained from Trevigen (Cat. No. 3455-096-02). Cell migration and invasion was quantified using Calcein-AM (Biotium #80011-3), in a 3-day protocol. On Day1, the appropriate transwell inserts were coated with 1x Basement Membrane Extract, and incubated at 37^o^ C overnight in 5% CO_2_. Some of the wells were uncoated for migration assays. On Day2, 4×10^4^ cells were plated in each transwell insert, and stimulated with FBS attractant (blank wells were used for background subtraction). On Day 3, a standard curve was established for each cell line, which allowed conversion of fluorescent values to number of cells, and detection of cells which passed through the membrane was performed with a fluorescence plate reader (Synergy HT, with KC4 software fro Bio-Tek Instruments) with excitation filter at 485 nm and emission filter at 520 nm.

#### Acute induction of oxidative stress

Cells were washed with PBS and then incubated with 100 µM DCFH_DA in medium in 5% CO_2_ at 37° for 30 min. After DCFH-DA was removed, cells were washed and treated with a series of concentrations of H_2_O_2_ in medium (0–400 mM). Fluorescence of cells was immediately measured using a plate reader (485nm for excitation, 530nm for emission). Data points were taken every 30min for 2hr. Fluorescence was essentially linear over the concentration range tested, and 200 mM was selected. The nonionic, nonpolar DCFH-DA (2′,7′-dichlorofluorescin diacetate) crosses cell membranes and is hydrolyzed enzymatically by intracellular esterases to non-fluorescent DCFH. In the presence of reactive oxygen species (ROS), DCFH is oxidized to highly fluorescent dichlorofluorescein (DCF). Therefore, the intracellular DCF fluorescence can be used as an index to quantify the overall ROS in cells.

#### MicroRNA array analysis

2 independent analyses were performed for all 4 MCF10A/siRNA cell lines, as well for MCF10A vector control and the over-expressing MCF10A/HtrA1 cell lines. 5 x 10^4^ cells were plated and cultured as described under Cell Growth Studies, and cells were harvested after day 4. Analyses were performed using the Illumina v2 Human MicroRNA Assay Beadchip assay (Illumina, San Diego, CA) in the PSU-COM Functional Genomics Core Facility. RNA quality and concentration was assessed using an Agilent 2100 Bioanalyzer with RNA Nano LabChip (Agilent, Santa Clara, CA). cRNA was synthesized from 200 ng of total RNA according to manufacturer’s instructions. The method targets specific sequences with sets of oligonucleotides which are extended, and labeled during PCR amplification. miRs were polyadenylated using Poly-A Polymerase (PAS, Illumina). The introduced poly-A tail was then used as a priming site for cDNA synthesis. The primer used in cDNA synthesis was biotinylated and contained a universal PCR primer sequence. The biotinylated cDNA was annealed to miR-specific oligonucleotides that correspond to all of the targeted microRNAs (1146 human microRNAs). The resulting single-stranded fluor-labeled PCR product was hybridized on the beadchip overnight with a temperature ramp from 60°C to 45°C. Following hybridization, beadchips were washed and scanned with a BeadArray Reader (Illumina, San Diego, CA). A project was created with resultant scan data imported into Genome*S*tudio 1.0 (Illumina). Results were exported to GeneSpring 7.3 (Agilent Technologies). Measurements less than 0.01 were then set to 0.01, arrays normalized to the 50^th^ percentile, and individual genes normalized to the median of controls.

#### Gene expression microarray analysis

3 independent microarray analyses were performed for each of the cell lines examined. 5 x10^4^ cells were plated and cultured as described in Cell Growth Studies, and cells were harvested after day 4. Microarray analyses were performed using the Illumina Human Whole Genome Beadchip (Illumina, San Diego, CA) in the PSU-COM Functional Genomics Core Facility. RNA quality and concentration was assessed using an Agilent 2100 Bioanalyzer with RNA Nano LabChip (Agilent, Santa Clara, CA). cRNA was synthesized by TotalPrep Amplification (Ambion, Austin, TX) from 500 ng of RNA according to manufacturer’s instructions. T7 oligo (dT)-primed reverse transcription was used to produce first strand cDNA. cDNA then underwent second strand synthesis and RNA degradation by DNA Polymerase and RNase H, followed by filtration clean up. *In vitro* transcription (IVT) was employed to generate multiple copies of biotinylated cRNA. The labeled cRNA was purified using filtration, quantified by NanoDrop, and volume-adjusted for a total of 1.5 µg/sample. Samples were fragmented, and denatured before hybridization for 18 hours at 58°C. Following hybridization, beadchips were washed and fluorescently labeled. Beadchips were scanned with a BeadArray Reader (Illumina, San Diego, CA). A project was created with resultant scan data imported into GenomeStudio 1.0 (Illumina). Results were exported to GeneSpring 7.3 (Agilent Technologies). Measurements less than 0.01 were then set to 0.01, arrays normalized to the 50^th^ percentile, and individual genes normalized to the median of controls.

Standard FDR [86], [87] and Sidak [88] corrections were used to further analyze microarray data.

### Silhouette Measure to Determine the Optimal Number of Clusters

To determine the optimal number of clusters we used the *Silhouette width* index [89]. This Silhouette width for each point is a measure of how close that point is to the points of its own cluster compared to points in other clusters: s(i)  =  b(i) – a(i)/max{a(i),b(i)} with b(i)  =  mink{B(i,k)} where,

s(i)  =  Silhouette width for each point i

a(i)  =  The average distance between point *i* and points of its own cluster

B(i,k)  =  The average distance between point i and points of another cluster k b(i)  =  The minimum of the average distance between point *i* and all other points in other clusters

The Silhouette width value ranges from −1 to 1.

A value close to 1 means that the sample is well clustered.
A value close to zero means that it could be assigned to another cluster as well.
A value close to −1 means that that the point i has been most likely misclassified.

We can compute the average Silhouette width for each cluster and for the entire dataset. The optimal number of clusters would be the one that maximizes the overall Silhouette width for the entire dataset.

#### Statistical analyses

For all the quantitative real-time PCR, cell growth rate, migration and invasion, phosphorylated-γH2AX cell staining assays, a Student’s paired *t* test was used to determine statistical significance (Microsoft Excel). Results are expressed as means ± SE. Values were considered as statistically significant if *P*<0.05.
